# Cardiac regeneration: epicardial mediated repair

**DOI:** 10.1098/rspb.2015.2147

**Published:** 2015-12-22

**Authors:** Teresa Kennedy-Lydon, Nadia Rosenthal

**Affiliations:** 1 National Heart and Lung Institute, Imperial College London, London, UK; 2 Australian Regenerative Medicine Institute, Monash University, Melbourne, Victoria, Australia; 3 The Jackson Laboratory, Bar Harbor, ME, USA

**Keywords:** regeneration, epicardium, cardiac stem cells

## Abstract

The hearts of lower vertebrates such as fish and salamanders display scarless regeneration following injury, although this feature is lost in adult mammals. The remarkable capacity of the neonatal mammalian heart to regenerate suggests that the underlying machinery required for the regenerative process is evolutionarily retained. Recent studies highlight the epicardial covering of the heart as an important source of the signalling factors required for the repair process. The developing epicardium is also a major source of cardiac fibroblasts, smooth muscle, endothelial cells and stem cells. Here, we examine animal models that are capable of scarless regeneration, the role of the epicardium as a source of cells, signalling mechanisms implicated in the regenerative process and how these mechanisms influence cardiomyocyte proliferation. We also discuss recent advances in cardiac stem cell research and potential therapeutic targets arising from these studies.

## 1. Introduction

An emerging concept in cardiovascular biology is that the mammalian myocardium has the potential to regenerate; however, the repair processes are insufficient to address the extensive damage caused by a cardiac insult such as myocardial infarction (MI). MI culminates in severe ischaemic damage to the surrounding tissue with, in some cases, the loss of up to a billion cardiomyocytes (CMs) [1,2]. This results in invasion of immune cells and myofibroblasts promoting scar formation [3,4]. With many advances in medical treatment, the mortality rate post-MI has decreased [5,6]; however, this has resulted in a rise in the number of patients that now present with heart failure [7–9]. For these reasons, research is now focused on enhancing the existing repair processes to improve cardiac function and to prevent the advent of heart failure. One approach is to study animal models that undergo full cardiac regeneration and extrapolate these observations to the adult human heart.

## 2. Models of cardiac regeneration

The zebrafish, a member of the teleost family, is noted for its cardiac regenerative capacity, and as a result is exploited throughout the field. Structurally, the zebrafish heart differs from that of mammals insofar as it is a two chamber organ, consisting of one atrium and one ventricle [10,11]. As the zebrafish heart consists of numerous small vessels for oxygen uptake rather than the large coronary vasculature typical of mammalian hearts, methods for inducing cardiac injury are limited to apex resection, cryoprobe injury or genetic ablation [12–14]. Following resection, a fibrin clot forms, providing a platform for proliferating CMs [15,16]. Remarkably, as demonstrated using a transgenic model which is deficient in ventricular myoctes, atrial CMs undergo transdifferentiation and replace lost ventricle CMs, although it should be noted that this mechanism is lost by the age of four months [17]. Interestingly, the medaka, a close relative of the zebrafish, lacks the ability to regenerate following resection. Injured medaka hearts present with persistent collagen deposition, lack of vascularization and a limited number of proliferating CMs [18], demonstrating the variation in evolutionary conservation of regeneration even within closely related species.

Similar to zebrafish, axolotls are also capable of replacing tissue following apical amputation [19,20]. Here again the repair process begins with the formation of a blood clot followed by CM cell cycle re-entry. The newt also retains the ability to regenerate the heart post-resection; however, not all CMs re-enter the cell cycle [21,22]. Moreover, replenishment of CMs in the newt is initiated by tenascin-C, a component of the extracellular matrix (ECM) [23], rather than infiltration of resident progenitor cell populations such as is the case with other models of regeneration. For this reason, the newt heart is an attractive model for understanding the mechanism(s) involved in CM proliferation and, more importantly, inhibitors of this process.

Recent studies on the ability of the neonatal murine heart to regenerate following injury would suggest that the mammalian heart does in fact harbour regenerative capabilities, albeit limited to immature stages: murine hearts undergo full regeneration post-MI from post-natal day 1 (P1), with loss of regenerative capacity evident by P7 [24,25]. Irrespective of species differences, a common thread between the aforementioned *in vivo* models is their ability to maintain CM proliferation throughout adulthood. This mechanism is largely lost in adult mammalian hearts, and while there are reports of proliferating CMs [26–28], there are too few to make any impact on the repair process.

## 3. Epicardial signalling

A common theme with successful models of regeneration is their ability to facilitate CM proliferation and the perfusion of injured tissue via neovascularization, of which the epicardium plays a central role. The epicardium contributes to heart development through secretion of a number of factors and controlled expression of developmental genes that have been shown to be instrumental to normal heart development. Collectively, these epicardial makers identify a cell population that is capable of giving rise to cell lineages that are deemed to be epicardially derived and therefore of mesothelial descent, which goes to explain how once activated and under optimum conditions the epicardium can give rise to fibroblasts, smooth muscle cells and endothelial cells. In addition, the epicardium has been described as a source-pool for cardiac stem cells (CSCs) [29–32]. These unique features of the epicardium and their respective signalling mediators will be discussed in the following paragraphs.

### (a) Wilms tumour gene

Wilms tumour gene 1 (Wt1) is a transcription factor that is expressed in many tissues, including the urogenital system, spleen, brain, spinal cord, mesothelial organs, diaphragm, limb, proliferating coelomic epithelium, epicardium and subepicardial mesenchyme, during development [33,34]. Disruption of Wt1 activity results in developmental abnormalities, and Wt1^−/–^ mice are embryonic lethal at embryonic day 12.5 (E12.5), with heart failure being one of the contributing factors to their early demise [33]. In the adult mammalian heart, Wt1 is reactivated following MI [35,36], although the specificity of Wt1^+^ cells epicardial origin has been questioned. Wagner *et al.* identified Wt1^+^ endothelial and vascular smooth muscle cells in the infarct and border zone, and attributed observed de novo neogenesis to Wt1^+^cells activated by hypoxia [35]. In addition, Duim *et al.* have recently identified a population of Wt1^+^ endothelial cells that undergo proliferation in a hypoxic environment both *in vitro* and *in vivo* following MI [37]. In contrast, Zhou *et al.* attribute the beneficial impact of Wt1^+^ cells in infarcted hearts to paracrine signalling and secretion of proangiogenic factors, rather than to a rise in Wt1^+^ endothelial cells [36]. Despite these conflicting reports, there is substantial evidence to show that Wt1 is activated following injury and, once activated, contributes to angiogenesis. Fate mapping studies have revealed Wt1 is expressed in endothelial cells, which points to its unsuitability as an exclusive epicardial marker. Although initially hampered by the lack of a definitive lineage trace model [38], collectively these *in vivo* studies demonstrate the beneficial impact of reactivating Wt1 in the adult epicardium, and establish a link between Wt1 expression and vascular formation.

### (b) Thymosin β4

The G-actin sequestering peptide thymosin β4 regulates actin-cytoskeletal organization necessary for cell motility, organogenesis and other cell functions. Following MI, thymosin β4 has been shown to induce epicardially derived cells (EPDCs) to form vascular precursors and prompt neovasculogenesis [39,40]. Rossdeutsch *et al.* identified thymosin β4 expression in embryonic endothelium, and demonstrated that it promotes mural cell maturation and differentiation, and embryos lacking thymosin β4 were subjected to severe haemorrhaging (which in some cases proved to be lethal) [39]. A follow up study by Smart *et al.* has demonstrated that the addition of exogenous thymosin β4 can enhance cardiac repair by directing Wt1^+^ cells to undergo cardiomyogenesis [41], confirming earlier findings [42]. The proangiogenic effects of thymosin β4 in the adult heart were confirmed in a study by Shrivastava *et al.* [43] where mice were given a systemic injection of thymosin β4 immediately following MI injury, resulting in an increase in vessel density at the border zone and remote zone, and demonstrating the global effects of thymosin β4. *In vitro* data from the same study attributed the proangiogenic effects of thymosin β4 to protein kinase C signalling and confirmed that thymosin β4 can reactivate epicardial embryonic genes, including Wt1 and Tbx18 [43].

In addition, Rui *et al*. [44] have extended the cardiac regenerative capacity of neonatal mice by pre-treatment with thymosin β4. Here, neonatal mice were subjected to daily intraperitoneal injections of thymosin β4 from P1 to P7; apical resection was performed at P7, and 19 days post-injury neonatal hearts displayed complete regeneration, which was attributed to an increase of WT1^+^ cells. Notably, post-injury mice continued to receive intraperitoneal injections of thymosin β4 on alternate days until the study was terminated, potentially affecting the validity of the claimed extension of the neonatal window.

Conversely, a report by Banerjee *et al.* failed to document any effect of thymosin β4 on mural cell migration, angiogenesis or embryo development. In this study, thymosin β4 knockout models were not embryonic lethal and a lack of thymosin β4 had no impact on vascular development [45]. These contrasting results have been attributed to variation of environment or genetic background, resulting in differing compensatory mechanisms in response to the loss of thymosin β4 between strains [46]. Contrasting data on mural cell activity has also been attributed to the different time points at which mural cell activity was assessed between these studies. Since thymosin β4 seems to have a global impact on the heart, particularly in response to injury, teasing out a specific role for this molecule is likely to generate a number of contrasting views. Future investigations into the role of cardiac thymosin β4 are eagerly anticipated.

### (c) Follistatin-like-1

A recent study has demonstrated the potential clinical application of follistatin-like-1 (Fstl1) as a catalyst for CM replenishment [47]. Fstl1 is a secreted glycoprotein and has been described as a cardiokine (cardiac secreted protein that can be used as a biomarker of cardiac dysfunction). Fstl1 belongs to the follistatin protein family, which mediate their effects by binding to transforming growth factor β (TGFβ) [48]. It is expressed in the developing heart and in the epicardium of the adult heart. Following either MI or ischaemic reperfusion, Fstl1 is upregulated in murine models [49]. In addition, Fstl1 has been detected in circulation in patients with acute coronary syndrome and protein levels are increased in failing hearts, hence its suitability as a biomarker of cardiac dysfunction [50,51]. The cardioprotective effects of Fstl1 have been associated with suppression of apoptosis and inflammation [49,52]. Fstl1 activates AMPK signalling, which decreases expression of proinflammatory genes in both macrophages and CMs *in vitro*, and administration of Fstl1 to damaged myocardium reduces the expression of proinflammatory mediators in areas of ischaemic damage *in vivo* [52].

In an exciting extension of these observations, epicardial patches seeded with Fstl1 were sutured onto the mouse epicardium immediately following MI injury, which resulted in CM cell cycle re-entry and division of existing CMs [47]. Only epicardially derived FStl1 could initiate cell division of CMs, whereas myocardial Fstl1 did not evoke CM cell division. This effect was specific to naive CMs (i.e. adult ventricular CMs did not undergo cell division in response to Fstl1). Intriguingly, this study shows that the source of Fstl1 determines its regenerative (epicardial) versus cardioprotective (myocardial) potential.

Taken together, these studies shift the focus from Fstl1 as a biomarker to a viable therapeutic that is ripe for clinical exploration.

### (d) T-box genes

Several T-box family transcription factor genes have been implicated in cardiac regeneration. Studies have demonstrated that epicardially derived Tbx18^+^ cells give rise to CMs [53,54], although it should be noted that these findings were later disputed as Tbx18 was subsequently detected in the myocardium [55]. Thus claims that new CMs solely originate from the epicardium cannot be substantiated. Nevertheless, Tbx18 has been shown to be upregulated following MI with a similar expression pattern to Wt1 [36]. Moreover, Tbx18 has been shown to be upregulated following priming with thymosin β4 [41].

Interestingly, Tbx20 has been identified in cardiac fibroblasts as a key regulator of myofibroblast differentiation [56]. Moreover, Tbx20 has been shown to regulate scar formation following MI; *in vivo* models devoid of fibroblasts expressing Tbx20 develop thicker scars [56]. This study highlights a unique role for cardiac fibroblasts in managing scar formation following MI.

### (e) C/EBP

A study by Huang *et al.* has identified the C/EBP transcription factor family to be key for neutrophil-mediated activation of the epicardium following MI [57]. Disruption of C/EBP signalling in the epicardium blunted the inflammatory response following ischaemic reperfusion injury, resulting in inhibition of invading neutrophils, the net result of which was reduced scar formation. This study offers key insights into myocardial scar and epicardial communication, highlighting the fine balance between inflammation and repair, and defining a novel role for the epicardium as a modulator of the inflammatory response: an interesting hypothesis worthy of further investigation.

### (f) Hypoxia-inducing factor

Hypoxia-inducing factor-1α (HIF-1α) is upregulated following MI and increases vascular endothelial growth factor (VEGF) expression in ischaemic tissues [58,59]. Furthermore, myocardial overexpression of HIF-1α increases vascular density, reduces scar formation and improves cardiac function [60]. Following MI, HIFs are expressed by numerous cell types, including CMs, interstitial cells and endothelial cells [61]. Notably, HIFs have been shown to occupy distinct sites within the epicardium that correspond with coronary vasculogenic patterning [62]; this may be in conjunction with HIF-mediated expression of Wt1 [63]. Furthermore, constituent expression of HIF-1α in avian epicardium via a viral vector showed that although EPDC epithelial mesenchymal transition (EMT) was enhanced, migration of EPDCs into the myocardium was impaired [64]. Thus, while HIF-1α may be important for driving vasculogenesis, it can also serve as a negative regulator of EPDC migration.

### (g) Retinoic acid

Epicardially derived retinoic acid (RA) synthesizing enzyme 2 (Raldh 2) is responsible for CM proliferation and differentiation, as well as ventricular maturation and angiogenesis [65]. Furthermore, Raldh 2 plays an important role in cardiac development; indeed, mice lacking retinoid X receptors die mid-gestation due to detachment of the epicardium [66,67]. In adult zebrafish, endocardial Raldh 2 has been shown to be intrinsic to repair of the heart following resection [68]. Inhibition of RA receptors or RA-degrading enzyme blocked the regenerative response of the zebrafish heart [68]. Furthermore, a lack of Raldh 2 expression in the endocardium of the medaka is suggested as a key factor for their inability to regenerate [18]. In murine hearts, RA signalling is reactivated following MI, and may play an important role in the repair and remodelling processes post-ischaemic injury due to its anti-proliferative effects on cardiac fibroblasts [69]. The use of RA as an anti-proliferative treatment following MI is an exciting prospect for future therapeutic interventions; indeed, RA derivatives have been used to suppress proliferative diseases such as prostate, lung, breast, skin, ovarian, bladder and oral cancer [70]. Further investigations are warranted to elucidate the contribution of RA signalling to repair of the mammalian heart.

### (h) Growth factors

Migration of EPDCs to the myocardium is key to regenerating damaged myocardial tissue; therefore manipulation of myocardial fibroblast growth factor (FGF) to epicardial FGF receptor 1 (FGFR1) presents an interesting model for myocardial mediated self-repair. Epicardial FGFR1 is upregulated following activation of myocardial FGF [71]. In addition, overexpression of myocardial FGF results in increased EPDC expression of FGFR [72]. Furthermore, the FGF family has also been implicated in regeneration of the zebrafish adult heart. Here, FGF 17b has been localized to the myocardium, while FGFR2 and FGFR4 were found in the epicardium [73]. These studies suggest that the expression of FGF in the myocardium can regulate FGFR expression in the epicardium—the result of which is epicardial cells undergoing EMT [72,73]. Interestingly, studies on explant hearts show that activation of FGFR is important for the migration of proepicardial cells to the subepicardium and myocardium [72].

Similarly, there is evidence to support involvement of growth factors such as platelet-derived growth factor (PDGF) and TGF*β* and VEGF in myocardial to epicardial communication. As with FGFs, PDGF and VEGF factors promote EMT and favour the fate of vasculogenesis [74,75], while TGF*β* favours smooth muscle cell differentiation [76].

The other major invading cell type following MI are macrophages, which are an abundant source of cytokines, chemokines and growth factors including insulin-like growth factor 1 (IGF-1). Several studies have demonstrated the beneficial impact IGF-1 has on the heart following MI [77–79]; forced cardiac-specific overexpression of IGF-1 improves cardiac function and reduces scar formation post-MI [77]. Macrophages also secrete proangiogenic factors including VEGF and TGF*β*, and have been shown to contribute to angiogenesis post-injury [80–82]. Increasingly, macrophages are being recognized for more than their ability to invade and phagocytose debris, and indeed their ability to secrete cytokines and chemokines at injured sites makes them an attractive prospect for clinical interventions. Recently, macrophages have been implicated in the neonatal regenerative response [83]. Although there are difficulties in determining which populations are contributing to the repair process (resident versus circulatory), their role in the repair of the mammalian heart merits further investigation.

## 4. Epicardial signalling: clinical application

**Figure 1.**
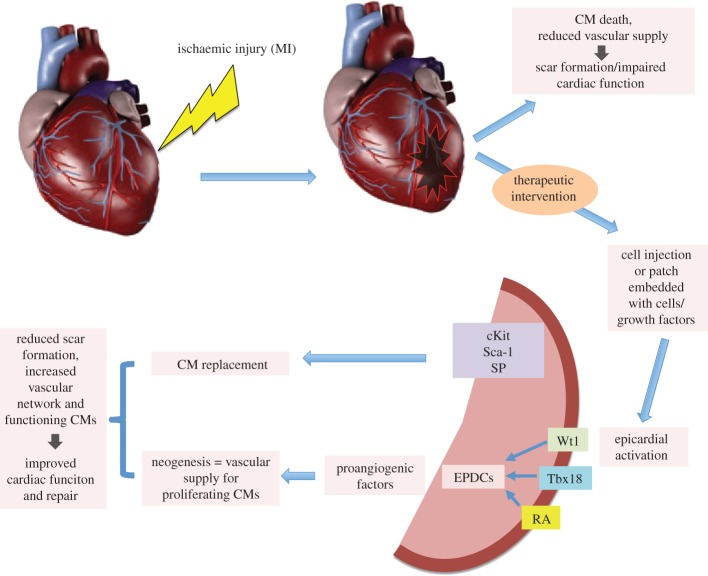
Schematic of proposed repair following therapeutic intervention. Following ischaemic injury, the epicardium is activated following either cell injection or application of a patch loaded with cells or growth factors, switching on fetal genes Wt1, Tbx18, RA and progenitor pools cKit Sca-1 and SP, which signal back to damaged myocardium through the secretion of proangiogenic factors to evoke neogenesis. Activation of progenitor pools promotes CM proliferation. The net result is reduced scar formation, increased vascular network and a new CM population. CM, cardiomyocyte; Wt1, Wilms tumour gene; RA, retinoic acid; Tbx18, T box gene 18; EPDC, epicardially derived cell. (Online version in colour.)

Providing an adequate vascular supply that can meet the metabolic demands of CMs and facilitate clearing of the debris from the immune response is a critical step towards restoring cardiac function. Therefore, the ability to initiate angiogenesis is key to the repair/regenerative processes. EPDCs have the ability to differentiate to coronary lineages and the majority of paracrine signalling from the epicardium is proangiogenic (figure 1). Given that endogenous repair mechanism(s) in the adult favour angiogenesis, methods to improve reperfusion should be a primary focus of regenerative medicine, as replacement of CMs will not be successful without the necessary infrastructure to meet the demands of these highly metabolic cells.

To this end, the use of biomaterials such as hydrogels or patches could have a significant impact. Here, cells are incorporated within a gel or patch which can then be injected or applied to the epicardium [84–87]. In theory, cells can migrate to the injured myocardium-secreting paracrine factors to enhance the repair process. This approach may also have the additive effect of activating the epicardium to secrete key factors such as Wt1, RALDH, thymosin β4 or Fstl1. Indeed, a recent study using an IGF-1-loaded fibrin patch post-MI in a porcine model reported improved cardiac function [88]. The use of cardiac patches also has the added benefit of providing mechanical support to the weakened epicardium post-ischaemic injury [89]. Although this approach is still in its infancy, and considerations such as the immune response and potential rejection of these materials need to be fully assessed, it does seem an intriguing therapeutic avenue.

## 5. Cardiac stem cells

The heart contains a number of distinct CSC populations; EPDC, stem cell antigen-1 (Sca-1), c-Kit, cardiosphere-forming CSCs and side population (SP) progenitors. Although a variety of CSCs exist, they all share a common feature: their reported ability to give rise to all cardiac lineages. Here, the epicardium again plays a significant role. The epicardium itself is derived from mesothelial cells, thus it is reasonable to assume that it can give rise to progenitor cell types [29,42,54,90,91]. Furthermore, the reactivation of embryonic cell markers post-MI such as Wt1 demonstrates that the epicardium retains signature gene expression patterns that are intrinsic to fetal growth and development, and therefore potentially maintains the necessary machinery for repair. Moreover, studies have shown that EPDCs are capable of undergoing EMT in adult hearts giving rise to smooth muscle cells [90].

One CSC population that has divided opinion within the field is that of cKit^+^ cells, with recent investigations disputing earlier findings that demonstrated cKit^+^ cells give rise to CMs. Orlic *et al.* first reported that bone marrow-derived cKit^+^ cells regenerate the myocardium following MI and reported cKit^+^ cells as CSCs [92]. This was later disputed by Murry *et al.*, who showed that cKit^+^ cells do not give rise to CMs [1]. Later studies confirmed these observations and went on to claim that CMs are derived from pre-existing CMs [27]. However, a study by Ellison *et al.* [32] argued that cKit^+^ cells are not only necessary but are sufficient to mediate repair to the damaged myocardium. Conclusively, a thorough investigation using genetic lineage-tracing models demonstrated that cKit^+^ cells do indeed give rise to CMs in both uninjured and injured tissue, but at a much lower rate than previously reported [93]. With this question outstanding, the field currently errs on the side of caution with regard to the potential of cKit^+^ cells as a pool of CM precursors.

Irrespective of these discrepancies, ongoing studies are investigating cardiospheres, essentially clusters of proliferating mesenchymal/stromal cells that express cKit. This unique cluster formation is thought to enhance ‘stemness' [94]. Interestingly, as cardiospheres diverge to a monolayer, they lose their cKit expression. Given that cluster formation recapitulates a stem cell-like microenvironment, it is not unreasonable to assume that cKit plays a role in this structure formation. Post-MI, cardiosphere injection has been shown to be more effective in improving cardiac function than injections of monolayer cells [95]. The therapeutic benefits of cardiospheres over cell monolayers is attributed to enhanced expression of ECM and adhesion molecules, as post-MI these clusters are retained at the site of injury with greater potency than injection of cells from a monolayer [94]. Thus, injection of multicellular spheres may prove to be the way forward for successful stem cell treatment.

Other CSCs have been identified based on their expression of Sca-1. Sca-1 is a ubiquitous stem cell marker associated with haematopoietic stem cell lineages. In the heart, Sca-1 expression is used to distinguish CSCs from differentiated cells. The Sca-1^+^ population was first observed in endothelial cells that also expressed cardiac transcription factors such as myocyte enhancer factor 2 (Mef 2) and GATA 4 [96]. Moreover, these cells were shown to give rise to CMs *in vitro* and *in vivo* [96,97]. Other studies have reported that Sca-1^+^ cells can give rise to CMs, endothelial cells and fibroblast cells [31,98]. Recent studies have identified a Sca-1 population derived from endothelial lineage that can migrate and give rise to CMs [99]. Notably, a Sca-1 gene analogue has yet to be found in the human genome and therefore caution should be taken when extrapolating these findings to the human heart. However, the murine Sca-1 antibody has been used to isolate Sca-1^+^ cells from the human heart, presumably cross-reacting with a related peptide, and these populations were also found to express GATA 4 and Mef 2 [100].

Further studies have led to the discovery of another progenitor population, referred to as SP. These cells are characterized by their unique ability to exclude Hoechst dye via the ATP-binding cassette transporter [101]. The cardiac SP population which is Sca-1^+^ and CD31^−^ has been shown to be immunophenotypically distinct from bone marrow-derived SP population, and is capable of self-renewal and giving rise to functionally mature CMs [102].

A recent study has demonstrated that PDGR*α* is necessary for the clonogenicity of SP cells and proposes that SP cells may be isolated based on PDGR*α* expression [103]. SP express cardiac transcription factors such as GATA 4 [102], thereby demonstrating their tendency towards cardiac-specific lineages. Post-MI, SP cells are initially depleted but are restored again by day 7, evidenced by a rise in the proliferation marker Ki67 [104]. This would suggest that SP cells contribute to the repair process following MI; however, their marked depletion at the critical 24 h period could have a profound effect on the course of the repair process. Perhaps if the SP population is somehow maintained following the initial injury, then the loss of CMs may be more readily appeased as the resident progenitor pools can readily replace lost CMs (figure 1).

## 6. Clinical application

While the evidence supports the existence of CSCs in the adult heart, these endogenous populations are often insufficient to repair the heart following MI. It is likely that the primary function of these populations is to support homeostatic maintenance under normal physiological conditions as opposed to mediating repair following severe injury such as MI. Nonetheless, if it were possible to sustain these populations, particularly 24 h post-MI, then such a repair process could positively contribute in the face of such significant CM depletion post-MI. Certainly, the capacity of CSCs to give rise to various cardiac lineages is certainly a feature that needs to be exploited. Accordingly, the method by which CSCs are delivered to the site of injury appears to be a major influence on their regenerative effect.

There have been many studies that demonstrate the regenerative effects from intramyocardial injection of stem cells (generally bone marrow-derived) following MI [105–107]. However, translating this approach to the clinic is hampered first by the difficulty in stem cell isolation and expansion. As previously discussed, many parameters are used to define CSCs, and isolating these cells is often a laborious task, with great care needed to ensure a pure population is obtained, although it has been demonstrated that SP cells can be effectively isolated based on PDGFR*α* expression as opposed to the current dye exclusion methods [103]. Second, the number of cells that are successfully engrafted is often much lower than those administered, so the mechanism by which stem cells exert their effects remains unclear, with varying reports of a paracrine effect [108,109], a result of fusion with endogenous cells [110] or transdifferentiation [111]. Third, recent data from clinical trials point to the ineffectiveness of stem cells, particularly bone marrow-derived stem cells, in treatment of cardiovascular disease, with many of these trials describing a positive outcome that was later found to be flawed by inaccurate reporting [112]. These setbacks in the clinical application of CSCs serve to reinforce the necessity for caution when interpreting results. However, recent clinical revelations should not impede preclinical advancements being made with CSCs. Initial studies with allogeneic cardiospheres using rodent models noted improved cardiac function, reduced scar formation and overall less damage to the myocardium [95]. The CADUCEUS (cardiosphere-derived autologous stem cells to reverse ventricular dysfunction) clinical trial reported similar findings and further clinical trials are being considered [113].

Given the diverse populations that make up the heart, perhaps a combination approach, such as that as demonstrated by Ye *et al*. [88] may prove to be a more worthwhile intervention to assist the repair process. Here, a trilineage engraftment consisting of CMs, ECs and SMCs derived from human-induced pluripotent stem cells (hiPSCs) was supported by pre-application of an IGF-1-loaded fibrin patch. The overall result was improved cardiac function with a greater engraftment rate than previously reported. This study was terminated four weeks post-cell injection; therefore, it is difficult to determine the long-term benefits. However, it does set a precedent for future *in vivo* approaches.

## 7. Conclusion

While cardiac regeneration appears to be easily achieved by lower vertebrates and amphibians, the adult mammalian heart struggles to function and mediate repair simultaneously. Until recently, the epicardium has been overlooked as being a key player in the repair/regenerative process. Today it is the focus of intense research, with many studies concentrating on the stem cell potential of this region of the heart. Although resident CSCs have been identified, the insufficiencies of endogenous stem cell populations to alleviate acute and chronic damage to mammalian cardiac tissue remain to be overcome. Therapeutically speaking, identifying methods to sustain stem cell populations during ischaemic damage may also prove fruitful in terms of replenishing lost CMs. Moreover, combination approaches exploiting the use of biomaterials and cell-based therapies are making remarkable advances. In addition, there have been various studies examining epicardial development, and these have aided our understanding of the adult mammalian heart injury response. Evidently, the epicardium is key to mediating repair; as a signalling powerhouse and cell reservoir, it is certainly equipped to do so. However, cardiac repair requires a speedy and robust approach, and although advances are being made we are still far from contriving an ‘off the shelf’ cure for the masses affected by heart failure.
